# CRISPR-Based Therapeutic Gene Editing for Duchenne Muscular Dystrophy: Advances, Challenges and Perspectives

**DOI:** 10.3390/cells11192964

**Published:** 2022-09-22

**Authors:** Guofang Chen, Tingyi Wei, Hui Yang, Guoling Li, Haisen Li

**Affiliations:** 1 Shanghai Key Laboratory of Maternal Fetal Medicine, Clinical and Translational Research Center of Shanghai First Maternity and Infant Hospital, Tongji University School of Medicine, Shanghai 201204, China; 2 Department of Anesthesiology, Ninth People’s Hospital, Shanghai Jiao Tong University School of Medicine, Shanghai 200011, China; 3 Shanghai Institute of Precision Medicine, Shanghai 200125, China; 4 HUIGENE Therapeutics Co., Ltd., Shanghai 200131, China; 5 Institute of Neuroscience, State Key Laboratory of Neuroscience, Key Laboratory of Primate Neurobiology, Center for Excellence in Brain Science and Intelligence Technology, Chinese Academy of Sciences, Shanghai 200031, China; 6 School of Medicine, Wayne State University, Detroit, MI 48201, USA

**Keywords:** DMD, CRISPR, dystrophin, gene therapy, double cut, single cut, base editing, prime editing

## Abstract

Duchenne muscular dystrophy (DMD) is a severe neuromuscular disease arising from loss-of-function mutations in the *dystrophin* gene and characterized by progressive muscle degeneration, respiratory insufficiency, cardiac failure, and premature death by the age of thirty. Albeit DMD is one of the most common types of fatal genetic diseases, there is no curative treatment for this devastating disorder. In recent years, gene editing via the clustered regularly interspaced short palindromic repeats (CRISPR) system has paved a new path toward correcting pathological mutations at the genetic source, thus enabling the permanent restoration of dystrophin expression and function throughout the musculature. To date, the therapeutic benefits of CRISPR genome-editing systems have been successfully demonstrated in human cells, rodents, canines, and piglets with diverse DMD mutations. Nevertheless, there remain some nonignorable challenges to be solved before the clinical application of CRISPR-based gene therapy. Herein, we provide an overview of therapeutic CRISPR genome-editing systems, summarize recent advancements in their applications in DMD contexts, and discuss several potential obstacles lying ahead of clinical translation.

## 1. Introduction

DMD, the most prevalent genetic muscular disease in man, is attributed to diverse mutations in the X-chromosome-resident *dystrophin* gene and affects approximately 1 in 3500 to 5000 newborn boys worldwide [1]. The *dystrophin* gene is the largest known human gene encompassing 2.6 million base pairs and contains 79 exons that encode a massive 427 kDa dystrophin protein [2,3]. The dystrophin protein, located underneath the sarcolemma, functions as a key mechanical anchor to connect the intracellular cytoskeleton to the inner surface of the muscle fiber membrane, maintaining sarcolemmal integrity and supporting muscle structure. Additionally, dystrophin plays a crucial role as the molecular scaffold to coordinate the assembly of numerous signaling molecules (e.g., nitric oxide synthase and ion channels), which work in concert to ensure the normal functioning of muscles [4,5]. However, the absence of dystrophin protein in DMD patients leads to muscle membrane fragility, myocyte necrosis, inflammatory infiltration, myocardial fibrosis, and progressive muscle weakness [2,6]. Due to the huge size of the *dystrophin* gene, more than 7000 pathological mutations, ranging from deletions, duplications, and point mutations to other small gene arrangements, have been discovered in DMD patients [7]. Most of these mutations (~75%) are intragenic deletions or duplications of one or multiple exons and primarily cluster into two hotspot regions between exons 2–22 and exons 43–55, whereas other small mutations (e.g., insertions and nonsense mutations) randomly occur throughout the *dystrophin* gene [7,8]. Pathologically, the majority of DMD mutations destroy the open reading frames (ORFs) or create a premature stop codon in the transcripts, which leads to aberrant translation and the generation of nonfunctional dystrophin. With the progression of DMD, dystrophin deficiency eventually results in the loss of ambulation, respiratory failure, cardiomyopathy, and premature death in early adulthood [6]. Unfortunately, although DMD is devastating, there exists no curative therapy for this lethal disease. At present, both corticosteroid and antisense oligonucleotide (AON) treatments are available for the mitigation of the symptoms of this disease [9,10,11], but they fail to remove the underlying genetic mutations from the *dystrophin* gene. It has been suggested that the current therapeutic approaches are offered mainly only for the alleviation of secondary manifestations of DMD, such as inflammation, fibrosis, mitochondrial dysfunction, impaired angiogenesis, or calcium dyshomeostasis [12,13,14,15,16,17,18,19]. Moreover, the long-term use of corticosteroids has been found to minimally ameliorate DMD phenotypes and cause many adverse effects, including growth delay and bone weakness [12,20,21]. Through the induction of the skipping of exon 45, 51, or 53 in dystrophin transcripts, four AON medicines have been approved to treat DMD patients with particular mutations in the exon 43–55 hotspot, but they usually restore dystrophin protein expression to less than 1% of the normal level after a year of continuous administration [22,23,24,25,26]. The broad application of AON medicines is restrained by various factors, including reduplicative administration, high cost, and poor delivery efficiency, particularly in the heart, probably due to their short half-lives [27,28]. Hence, there remains a great unmet need to develop innovative therapeutic strategies for correcting genetic mutations and restoring functional dystrophin generation in DMD individuals.

Based on its simplicity and precision, CRISPR-mediated genome engineering offers a promising therapeutic approach to restoring dystrophin expression and muscular functions in DMD individuals via eliminating pathological mutations at the genomic level [29,30]. Thus, a single administration of CRISPR genome-editing components can cure DMD in theory. The CRISPR system is composed of two major components, one CRISPR-associated (Cas) endonuclease and the other a single-guide RNA (sgRNA) complementary to the target genomic sequence [31,32]. Under the guidance of the sgRNA, Cas endonuclease directly binds to the target genomic sites adjacent to the protospacer-adjacent motif (PAM), creating DNA double-strand breaks (DSBs). In mammalian cells, these site-specific DSBs are typically resolved by endogenous cellular repair pathways, either non-homologous end joining (NHEJ) or homology-directed repair (HDR) [33]. The choice of DSB repair pathway depends on the cell type, cellular proliferation status, and the absence or presence of an exogenous DNA template. Thus far, CRISPR systems have been widely employed to correct diverse DMD-causing mutations not only in human DMD myoblasts and induced pluripotent stem cells (iPSCs) but also in preclinical DMD animal models such as mice, dogs, and pigs [34,35,36,37,38,39,40,41,42]. After the single systemic administration of Cas9 system components, both genomic editing and dystrophin restoration have been shown to persist for at least 18 months in the *mdx* mice harboring a point mutation in exon 23 and the DMD mouse model with an exon 44 deletion (ΔEx44) mutation [35,43,44], highlighting the durability of CRISPR therapeutics in dystrophic mice. Since adult human cardiomyocytes have an extremely low turnover rate over time [45], CRISPR gene therapy will most likely lead to lifelong benefits when used to treat DMD patients. However, clinical studies assessing the efficacy and safety of CRISPR gene therapy in DMD patients are still missing. Therefore, to facilitate the clinical translation of therapeutic CRISPR gene editing for DMD, more efforts are imminently necessary to address the multiple challenges, including but not limited to safe dosage, the in vivo delivery strategy, immunogenicity, and the extent of dystrophin restoration.

In this paper, we overview recent advances in the knowledge of CRISPR gene therapy for DMD and discuss several challenges in the clinical application of therapeutic CRISPR gene editing.

## 2. Dystrophin

There exist multiple different isoforms of dystrophin transcripts originating from seven unique promoters and alternative splicing [46]. Remarkably, the splicing patterns of 79 exons are highly conserved across vertebrate species [47], which aids the development of therapeutic medicines via evaluating the efficacy and mechanisms of potential medicines in preclinical DMD animals. Among these isoforms, the largest transcript driven by the promoter upstream of exon 1 is 14 kb in size and encodes for the 427 kDa dystrophin protein with 3684 amino acids. This full-length dystrophin is expressed in all skeletal muscles, heart, vascular and visceral smooth muscles, as well as some neural cells [48]. The dystrophin protein consists of four distinct functional domains in the following order: an actin-binding domain at the *N*-terminus, a central rod region with 24 successive spectrin-like repeats (SLRs), a cysteine-rich domain binding β-dystroglycan, and a *C*-terminal domain interacting with dystrobrevin and syntrophin [2,41]. Thus, dystrophin serves as an organizing center for the dystrophin-glycoprotein complex (DGC), linking the intracellular cytoskeleton to the extracellular matrix across the sarcolemma. Beyond dystrophin, other DGC components comprise an extracellular α-dystroglycan binding laminin-2, a trans-membrane β-dystroglycan, a membrane-resident sarcospan, and four trans-membrane sarcoglycans (α-, β-, γ-, and δ-sarcoglycan) [49]. Meanwhile, dystrophin has a direct association with neuronal nitric oxide synthase (nNOS) via two SLRs (R16 and R17) [5], localizing the nNOS near the sarcolemma and modulating nitric oxide signaling in muscle cells. It has been observed that DGC may associate with the proteins engaged in calcium homeostasis, such as plasma membrane calcium ATPase and calcium channels [50,51]. It is likely that the modulation of DGC on calcium signaling happens at the sarcoplasmic membrane level in dystrophic cells [50].

The deficiency of functional dystrophin results in the mislocalization of DGC components, dysregulation of calcium balance, abnormal nitric oxide signaling, mitochondrial dysfunctions, increased oxidative stress, defective energy metabolism, impaired autophagy, insufficient angiogenesis, and aberrant inflammation [51,52,53,54,55,56,57,58,59]. Intracellular calcium is known to be abnormally elevated in dystrophic cells owing to the activation of calcium channels in the sarcoplasmic membrane and the decreased calcium handling [51,52,53]. This elevation of cytosolic free calcium leads to an overload of calcium in the mitochondria and other organelles such as the endoplasmic reticulum, which in turn conduces to the augmentation of oxidative stress and the impairment of mitochondrial respiration and ATP production [54,55,56,60]. Consistent with these scenarios, defective energy metabolism is present in the dystrophic cells, partially evidenced by insufficient glucose utilization and fatty acid oxidation [57]. On the other hand, excessive cytosolic calcium and disturbed cellular homeostasis not only trigger the activation of calcium-sensitive proteases (e.g., calpains and phospholipase A2) but also promote the release of diverse cytokines and chemokines into the extracellular space. Such aberrant events provoke the infiltration of immune cells (e.g., macrophages and neutrophils) into dystrophic muscles [61], contributing to myofiber necrosis and muscle destruction. As time goes on, continuous cycles of muscle damage and regeneration progressively allow the replacement of dystrophic muscles with fibro-fatty connective tissue, accompanied by the loss of muscle mass and functions. It is noteworthy that muscle damage and membrane leakage are obligated to the unnatural elevations of serum creatine kinase and lactate dehydrogenase in DMD individuals [62]. These myriad dysfunctions have been well documented in the skeletal and cardiac muscles of both DMD patients and animal models [6,63,64,65].

Both the N terminus linking the actin filaments and the C terminus binding the DGC components are essential for dystrophin functions [66], whose mutations lead to a complete loss of dystrophin protein. On the contrary, the median domain having redundant rod repeats could be shortened and generates the internally truncated dystrophin forms with partial functions. It has been shown that a central domain with as few as 4 spectrin-like repeats enables truncated dystrophin to be partially functional [67]. Under natural conditions, internally shortened dystrophin proteins can be observed in patients with Becker muscular dystrophy (BMD) resulting from the in-frame mutations of the *dystrophin* gene [68]. Thus, BMD patients show relatively mild symptoms in general, and some patients may remain asymptomatic until later life [69,70]. Likewise, micro-dystrophin, a truncated but partially functional protein lacking redundant rod repeats, only contains a minimal number of functional domains and has been shown to ameliorate DMD symptoms in many preclinical animal models [71,72,73,74]. The adeno-associated virus (AAV)-mediated single administration of micro-dystrophin (e.g., SRP-9001, PF-06939926, and SGT-001) is still being evaluated in several clinical studies involving DMD boys (http://clinicaltrials.gov). Despite the tolerance of some DMD patients to micro-dystrophin therapy [49,75], the spatiotemporal expression of micro-dystrophin is actually modulated by an exogenous promoter within the AAV, which may lead to uncontrollable protein localization and level. On the contrary, the expression patterns of CRISPR-corrected dystrophin transcripts and proteins are controlled by endogenous promoters, representing an attractive advantage of CRISPR genome engineering.

## 3. CRISPR Systems

The CRISPR-Cas system was originally discovered as an adaptive defense system in bacteria and archaea against foreign viral pathogens [76,77,78] and has been harnessed for genomic editing in eukaryotic cells. Based on Cas type and number, six CRISPR systems (I–VI) are grouped into two distinct classes: the class 1 system, comprising types I, III and IV, needs multiple Cas effectors at once; the class 2 system, containing types II, V and VI, utilizes one single Cas endonuclease [79]. Most type-II Cas9 and type-V Cas12 proteins act on DNA and introduce the DSBs in a programmable manner, whereas type-VI Cas13 proteins cleave the RNA transcripts specifically via their RNA-targeting nuclease activity [32,80]. As for the manner of cleavage, Cas9 proteins with RuvC and HNH nuclease domains primarily create blunt-end DSBs in the protospacer sequence 3 nucleotides upstream of the PAM [76], while Cas12 variants possessing a single RuvC-like nuclease domain typically make the sticky ends with five-nucleotide overhangs in the PAM-distal regions of the protospacer [81]. It has been reported that *Streptococcus pyogenes* Cas9 (SpCas9) may cut DNA in a staggered orientation and leave a single-nucleotide overhang at the broken point [82,83]. Following DNA cleavage, the HDR pathway is activated to repair the DSBs in the presence of an exogenous donor template and introduces the desired modification into the genome at the target locus. Nonetheless, HDR is only active in the proliferative cells owing to its requirement of some proteins expressed in the S and G2 cell-cycle phases [84,85], so it has a very low efficiency (less than 2%) in post-mitotic cells such as cardiomyocytes and myofibers. On the contrary, the NHEJ machinery turns to fix the DSBs in the absence of an exogenous template and often generates small insertions or deletions around the broken site. Unlike HDR, NHEJ is functional throughout the cell cycle and operates at high efficiency not only in dividing cells but also in post-mitotic cells [86]. Thus, it is believed that NHEJ serves as the predominant repair pathway in most mammalian cells.

The diversification of Cas proteins is extremely remarkable in terms of the bacterial source, protein size, PAM sequence, suitable spacer length, and editing efficiency and specificity. For example, the most often used SpCas9 with 1368 amino acids specifically recognizes the tri-nucleotide PAM sequences of 5′-NGG or -NAG that are common in the human genome [76,77,87]. By comparison, *Staphylococcus aureus* Cas9 (SaCas9, 1053 amino acids) and *Campylobacter jejuni* Cas9 (CjCas9, 984 amino acids) utilize the longer but relatively restrictive PAM sequence of 5′-NNGRRT or 5′-NNNVRYM, respectively [88,89]. It should be noted that both SaCas9 (~3.2 kb) and CjCas9 (~2.95 kb) are much smaller than the genome packaging limit of AAV vectors (~4.7 kb) [90], enabling them to be efficiently delivered in vivo by conventional AAV vectors. In the cases of Cas12 effectors, *Lachnospiraceae bacterium* Cas12a (LbCas12a, 1228 amino acids) and *Acidaminococcus* sp. Cas12a (AsCas12a, 1307 amino acids) are more efficient and widely adopted than other Cas12 variants in mammalian cells via targeting the T-rich PAM of 5′-TTN [81,91,92]. Beyond these five Cas proteins, an enormous variety of Cas9 and Cas12 enzymes have been discovered and characterized from diverse bacterial species, including *Neisseria meningitidis* Cas9 (NmeCas9), *Streptococcus thermophilus* Cas9 (StCas9), *Francisella novicida* Cas9 (FnCas9), *Alicyclobacillus acidoterrestris* Cas12b (AacCas12b), *Bacillus hisashii* Cas12b (BhCas12b), *Oleiphilus* sp. Cas12c (OspCas12c), *Deltaproteobacteria* Cas12e (DpbCas12e), and *Planctomycetes* Cas12e (PlmCas12e) [79,93,94,95,96,97,98,99,100,101]. Additionally, many naturally occurring Cas proteins have been engineered to improve their PAM availability, enhance on-target efficiency, and/or reduce off-target activity [32,79]. Hitherto, a great deal of engineered Cas9 and Cas12 proteins with high targetability and specificity have already been made, such as eSpCas9, Spy-mac Cas9, HypaCas9, evoCas9, HeFSpCas9, xCas9, HiFiCas9, Sniper-Cas9, SpCas9-HF1, SpCas9-NG, FnCas9-RHA, SaCas9-KKH, AsCas12a-RVR, and enAsCas12a [102,103,104,105,106,107,108,109,110,111,112,113,114]. As new Cas9 and Cas12 variants unceasingly emerge, the toolkit of CRISPR gene-editing systems is getting expanded by both natural and engineered Cas proteins, thus offering more and more choices for CRISPR therapeutics.

An important engineering direction is to modify the nuclease domains of Cas enzymes to generate catalytically impaired nickase Cas (nCas) or catalytically inactivated dead Cas (dCas) variants while retaining their programmable DNA-binding ability [32,115]. In this regard, both nCas and dCas proteins have been fused with a cytidine deaminase or adenosine deaminase for single-base conversions or with an engineered reverse transcriptase for short insertions and deletions [116,117,118]. The fusion of nCas or dCas proteins with the cytidine deaminase APOBEC1 causes the precise transition from C•G to T•A base pairs [119], while the combination of nCas or dCas variants with the adenosine deaminase TadA accurately converts the targeted A•T base pairs to G•C [120]. Fusing an nCas effector with a reverse transcriptase can induce all base pair transitions, small insertions, and/or short deletions in a targeted and precise way [118,121]. The fusion protein between dCas9 and transcriptional activator VP160 could drastically increase the expression level of dystrophin homolog utrophin via targeting its promoters [122,123], which represents a promising compensatory approach for DMD treatment. Unlike conventional Cas enzymes, both nCas and dCas proteins are unable to introduce DSBs into the genome, making these nCas- or dCas-based CRISPR tools especially safe for genome editing and disease therapy.

## 4. CRISPR-Driven Therapeutic Strategies

The first demonstration of CRISPR gene-editing therapy is in the *mdx* mice through the direct zygote injection of SpCas9, sgRNAs, and single-stranded oligodeoxynucleotide (ssODN) [124]. Due to ethical issues and public policies, this germline editing approach is likely unavailable for DMD treatment in humans. Therefore, accumulating evidence instead validates the in vivo therapeutic benefits of CRISPR gene editing systems in postnatal DMD animals [36,37,39,40,43,44,125,126,127,128,129]. The leading strategies for the CRISPR-mediated therapeutic correction of DMD mutations are exon excision, exon skipping, exon reframing, exon knockin, base editing, and prime editing (Table 1 and Table 2). 

## 5. Double-Cut Exon Excision

**Figure 1:**
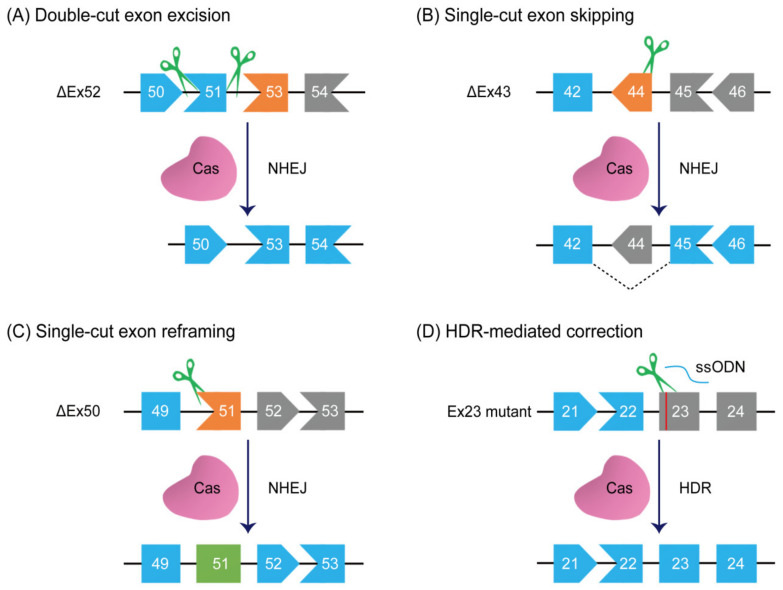

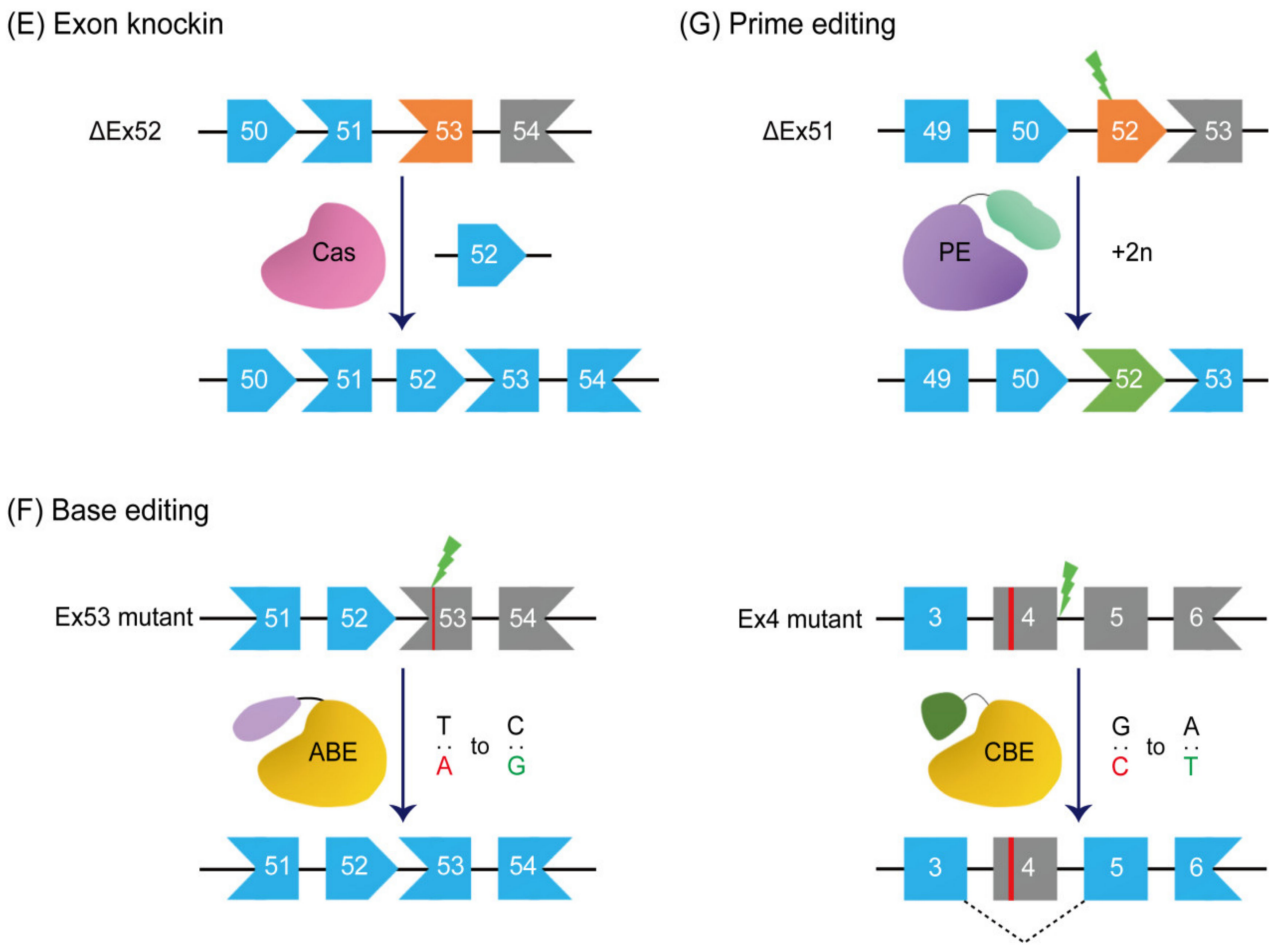
Therapeutic strategies for CRISPR-based genome editing. (**A**) Double-cut exon excision using two sgRNAs. In the end, exon 51 is removed from the genome by NHEJ machinery. (**B**) Single-cut exon skipping by disruption of the splice donor site. The exon 44 skipping induces the splicing of exon 42 to exon 45 at the mRNA level. (**C**) Single-cut exon reframing. Small insertions or deletions occurring in the exon 51 region can restore the reading frame with at least one-third probability. (**D**) Accurate mutation correction in the exon 23 by HDR pathway in the existence of a donor template. (**E**) HITI-mediated exon knockin. Exon 52 is precisely incorporated back into the genome by NHEJ machinery. (**F**) Base editing-driven correction. The ABE is used to treat a point mutation in the exon 53 via inducing A•T to G•C transition, whereas the CBE is deployed to mutate the GT at the splice donor site of exon 4, causing exon 4 skipping in the transcripts. (**G**) Prime editing-induced exon reframing. Prime editing can introduce all genomic modifications, such as single-nucleotide transitions, small insertions, and short deletions.

The removal of one or more exons by CRISPR gene editing applies to approximately 85% of all DMD patients bearing exon duplications, deletions, or point mutations. Two sgRNAs flanking either side of the mutant exons are designed to simultaneously cut the target genomic sites in the presence of Cas proteins, resulting in the complete excision of mutant exons (Figure 1A). Consequently, this kind of exon excision restores the dystrophin reading frame and the expression of functionally truncated dystrophin protein. It has been shown that this exon excision strategy is particularly suitable for correcting exon duplication mutations [122,125,150,151,152,153]. Exonic duplications are estimated to comprise 10–15% of all DMD mutations [152,164,165], making them the third most common cause of DMD. Under the guidance of two sgRNAs targeting a duplicated intronic region, SpCas9 precisely excludes a tandem duplication of exon 2. It repairs the expression of 7–11% of full-length dystrophin protein in human DMD myoblasts with an exon 2 duplication [151]. Likewise, the SpCas9 system has been employed to remove exon 18-30 duplication and induce full-length dystrophin restoration in human DMD myoblasts with exon 18-30 duplications (*Dup18-30*) [122]. Intravenously administrating AAV-SaCas9 system in DMD *Dup18-30* mice could ensure full-length dystrophin protein expression in cardiac and skeletal muscles ranging from 4% to 25% of the normal levels [125]. These levels of functional dystrophin restoration ameliorate dystrophic pathology, enhance muscle strength, and improve open-filed activity in SaCas9-corrected *Dup18-30* mice [125]. This observation aligns with the notion that as little as 3–14% of full-length dystrophin protein significantly benefits muscle functions [166,167]. Intriguingly, the elimination of a duplication event can be achieved readily with one sgRNA targeting the duplicated intronic region.

CRISPR-mediated exon excision is useful for correcting exonic deletion mutations, especially multi-exon deletions, in the *dystrophin* gene. This exon excision approach is supported in nature by the asymptomatic or mild symptoms of BMD patients with small in-frame deletions in the exon 45-55 mutation hotspot region [154]. It should be noted that the excision of the exon 45-55 hotspot region could be used as a treatment for more than 60% of DMD patients regardless of mutation type. For example, two different sgRNAs, one targeting intron 44 and the other targeting intron 55, are utilized to remove the entire exon 45-55 region in the presence of SpCas9, which in turn generates an internally truncated dystrophin protein in human DMD myoblasts with an exon 48-50 deletion (ΔEx48-50) [154]. This large excision of the exon 45-55 region efficiently repairs dystrophin protein expression, stabilizes the DGC complex, and improves membrane integrity in both cardiomyocytes and skeletal muscles from human DMD iPSCs harboring an exon 46-51 deletion (ΔEx46-51) mutation [38]. The restoration of functional dystrophin transcripts and protein following the excision of the exon 45-55 region has also been demonstrated in human DMD myoblasts with an exon 51 deletion (ΔEx51) mutation and a humanized DMD mouse model carrying the exon 45-deleted human *dystrophin* gene (hDMDΔ45/mdxD2) [130,155]. Notably, the efficiencies of the SpCas9 system when reframing the ΔEx51 mutation and restoring dystrophin expression are comparable to that of the AsCas12a system in human DMD ΔEx51 myoblasts and a patient-derived xenograft DMD mouse model [155]. Likewise, the combination of either SpCas9 or eSpCas9(1.1) with two sgRNAs targeting introns 43 and 54 is found to abscise the exon 44-54 region and generate a functional dystrophin protein in human DMD myoblasts with ΔEx48-50 or ΔEx45-52 mutation [156,157,158]. Intriguingly, the utilization of SaCas9 and two sgRNAs targeting exons 47 and 58 leads to the formation of a hybrid exon 47-58 lacking their internal large region and the expression of 360 kDa dystrophin protein in human DMD myoblasts with ΔEx49-50, ΔEx50-52, ΔEx51-53, or ΔEx51-56 mutation [40]. The systemic AAV delivery of SaCas9 components is further reported to restore functional dystrophin expression in the heart of humanized DMD mice with an exon 52-deleted human *dystrophin* gene (hDMDΔ52/*mdx*) [40]. Apart from the above multi-exon excision, the elimination of single exon 51 or 53 by the SpCas9 system has also been proved to repair the dystrophin reading frame in human DMD ΔEx48-50 or ΔEx45-52 myoblasts, respectively [154,156,157]. Moreover, the single systemic administration of SpCas9 components by AAV9 vectors in DMD ΔEx52 pigs restores dystrophin protein expression throughout muscle tissues, ranging from 12% to 54% of normal levels, which gives rise to the alleviation of muscle pathology, the improvement of skeletal muscle and cardiac functions, and the extension of porcine lifespan [39]. Notably, SpCas9-driven exon excision has been taken to handle the deletion mutation in the *N*-terminal exon 2-20 hotspot [159]. Three distinct excision approaches are designed to separately remove exons 3-7, 6-7, or 7-11 in human DMD iPSCs with an exon 8-9 deletion (ΔEx8-9) mutation. The exon 3-9 excision is the most effective strategy in restoring the contractility and calcium transits of DMD ΔEx8-9 iPSC-derived cardiomyocytes, whereas the exon 7-11 excision causes the minimal recovery of cardiomyocyte functionality due to the generation of a structurally unstable dystrophin protein [159]. When facing the mutations in *N*- and *C*-terminal domains, specific considerations need to be paid to retain the essential amino acid residues for functional dystrophin protein restoration.

Beyond exon duplication or deletion mutations, the exon excision strategy has been adopted to reframe the out-of-frame point mutations comprising ~27% of all DMD cases both in vitro and in vivo [34,35,36,37,43,126,127,128,129,131,160,168]. For example, AAVrh74-delivered SaCas9 and two sgRNAs targeting introns 20 and 23 in neonatal *mdx* mice are found to remove the exon 21-23 region with a nonsense mutation, prevent cardiomyopathy, and improve cardiac functions [43]. These benefits following systemic AAV-SaCas9 therapy can be sustained for up to 19 months without the occurrence of tumorigenicity and organ toxicity [43]. Likewise, the systemic administration of either SaCas9 or SpCas9 with two sgRNAs targeting introns 51 and 53 produces widespread dystrophin restoration in the cardiac, diaphragmatic and skeletal muscles of DMD mdx^4Cv^ mice harboring a point mutation in exon 53 [128,129]. The genomic editing efficiency of systemic AAV6-Cas9 treatment is stable in mouse cardiomyocytes rather than their skeletal muscles [129]. Intramuscularly administrating SpCas9 or SaCas9 components in DMD mdx^4Cv^ mice repairs dystrophin protein expression in up to 68% of skeletal myofibers, improves skeletal muscle structure, and boosts muscle forces [128]. Thus far, a single exon 23 excision using either SaCas9 or SpCas9 system has been validated in neonatal and adult *mdx* mice [34,35,36,37,127]. In neonatal *mdx* mice, systemically infused SaCas9 or SpCas9 components are capable of rescuing dystrophin protein expression in body-wide muscle tissues [34,36,37,127]. The local administration of the AAV-SaCas9 system in adult *mdx* mice leads to improved muscle morphology, ameliorated nNOS localization, and enhanced skeletal muscle force [36,127]. An interesting finding is that genomic editing efficiency and the extent of dystrophin restoration in the heart of systemically treated *mdx* mice increase as the mice age [37], which is probably due to the survival disadvantage of dystrophic cardiomyocytes.

Two major concerns limit the clinical application of double-cut exon excision at its current iterations. The first is its low editing efficiency, which may be attributed to the indispensability of two cooperative cutting across large genomic intervening regions. Another is the generation of diversely unpredictable genome modifications such as DNA inversion and AAV integration [37].

## 6. Single-Cut Exon Skipping and Reframing

Single-cut gene editing has emerged as a promising alternative strategy for the efficient and safe correction of diverse DMD mutations [41,169]. In this approach, one single sgRNA is designed to target the vicinity of the intron-exon boundary and splice signal sequences. Its utilization, together with the Cas enzyme, performs one single cutting. This single DSB is rejoined later by endogenous NHEJ pathways, introducing small insertions or deletions into the target loci. There are two repair outcomes: (1) exon skipping happens as small deletions abolish the splice consensus sites of out-of-frame exons (Figure 1B); (2) exon reframing occurs when an appropriate number of nucleotide deletions or insertions appear in the exonic region (Figure 1C). In theory, approximately one-third of single-cut editing events hold the promise to put the *dystrophin* gene back in the frame [42]. Indeed, SpCas9-mediated single cutting in exon 51 generates a large fraction of exon reframing events among all indels via preferentially inserting one single adenosine [138], while the reframing of exon 51 by the SaCas9-KKH system may even account for ~80% of all editing events [133]. Regardless of whether conducting exon skipping or exon reframing, the permanent restoration of the dystrophin reading frame and protein expression is eventually achieved in muscle cells. More than 80% of DMD patients are estimated to benefit from this therapeutic strategy [42]. Compared to double-cut exon excision, both exon skipping and exon reframing via single-cut gene editing possess many advantages, including but not limited to a high editing efficiency, low frequency of off-target events, and minimum genomic modifications.

Single-cut editing strategy is very efficient for reconstituting the dystrophin reading frame and expression in DMD mice and dogs with an exon 50 deletion (ΔEx50) mutation, representing one of the most common single exonic deletions in humans [133,135,138,139,140]. The AAV9-based intramuscular delivery of SpCas9 and one sgRNA in young DMD ΔEx50 mice and dogs can rescue dystrophin expression in nearly all skeletal myofibers and prevent the onset of skeletal muscle pathology [138,139,140]. In neonatal DMD ΔEx50 mice and young DMD ΔEx50 dogs, the systemic administration of SpCas9 components results in widespread dystrophin restoration throughout body muscles, improves the expression of DGC complex, repairs muscle structure and histology, and enhances muscle strength [138,139,140]. The restoration of dystrophin expression can be up to 92% of the normal level in the heart of systemically injected DMD ΔEx50 mice, in which ~21% of the genome-editing efficiency is yielded durably [138]. Likewise, a single intraperitoneal administration of the SaCas9-KKH system by AAV9 vector in neonatal DMD ΔEx50 mice has been shown to efficiently repair dystrophin expression in skeletal and cardiac muscles, ameliorate muscle structure and membrane integrity, and improve muscle functions such as contractility [133]. Beyond SpCas9 and SaCas9-KKH, both LbCas12a and AsCas12a systems have been shown to rescue dystrophin expression and increase mitochondrial number and oxygen consumption rate in human DMD ΔEx48-50 iPSCs-derived cardiomyocytes [133,142,150]. Recently, single-cut gene editing using the SpCas9 system has been extended to correct other single exonic deletions in human DMD iPSCs and DMD mouse models with ΔEx43, ΔEx44, ΔEx45, or ΔEx52 mutation [44,132,134,136]. The systemic AAV delivery of SpCas9 components in neonatal DMD ΔEx44 mice restores dystrophin expression in nearly all skeletal and cardiac muscles, consequently preventing muscle damage and improving muscle histology and force in the treated mice [44,132,136]. Notably, single-cut editing events in skeletal muscles of 18-month-old DMD ΔEx44 mice are around 15% more than in 1-month-old corrected ΔEx44 skeletal muscles [44], providing evidence for the lifelong benefits of single-cut gene therapy in DMD contexts.

Single-cut gene editing also provides an effective means of treating point mutations, small insertions, and short deletions in the exonic and intronic regions of the *dystrophin* gene [137,141,142,150]. Intramuscularly delivered CjCas9 and an sgRNA targeting the mutant exon 23 are sufficient to restore dystrophin expression and increase skeletal muscle force in the DMD mice with a 1-bp insertion or 14-bp deletion in exon 23 [137]. Likewise, SpCas9-driven single cutting in mutant intron 13 or 19 rescues dystrophin expression in the skeletal and cardiac muscles of WCMD or LRMD DMD canines bearing a small insertion in the intron 13 or 19, respectively [141].

Though single-cut editing is highly efficient in most DMD cases, its efficiency in genomic editing and dystrophin restoration varies dramatically from mutation to mutation. As an example, SpCas9 and one sgRNA targeting the splice donor site of exon 44 induces ~60% of dystrophin-positive myofibers in skeletal muscles of DMD ΔEx45 mice, but the same SpCas9 system merely restores dystrophin expression in ~36% of myofibers of a DMD ΔEx43 mouse model [134]. Meanwhile, the genome-editing efficiency, dystrophin restoration, and the extent of pathological amelioration depend on the dosage ratio of sgRNA to Cas9 protein [132,136]. Thus, the sgRNA sequence and its ratio to Cas protein must be well designed to achieve high therapeutic efficacy.

## 7. Exon Knockin

Despite its inefficiency in the post-mitotic cells, HDR-mediated gene editing has the capacity to produce full-length dystrophin protein regardless of DMD mutations. This therapeutic strategy is particularly useful for mutations in the essential *N*- and *C*-terminus regions of dystrophin. Thus far, the HDR-based knockin strategy has been exploited to handle either point mutations in DMD animal models or a single exonic deletion in human DMD iPSCs (Figure 1D) [124,128,142,143,144,170]. The intramuscular delivery of AAV6-encoded SpCas9, sgRNA and a donor template sequence in adult mdx^4Cv^ mice rescues dystrophin generation and improves skeletal muscle morphology, but its gene-editing efficiency is only about 0.18% [128]. In *mdx* mice, the application of 180-nt ssODN, sgRNA, and SpCas9 or LbCas12a by zygote injection could repair dystrophin expression in various muscle tissues at different restoration rates [124,142]. The gene-editing efficiencies of 17% to 41% in SpCas9-corrected mice and 8% to 50% in LbCas12a-treated mice probably come from the relatively high activity of HDR in zygotes [124,142]. Moreover, the SpCas9 system and a donor template have been harnessed to insert the missing human exon 44 back in DMD ΔEx44 iPSCs and generate full-length human dystrophin protein in their derivate cells [161]. Nonetheless, the HDR-based knockin strategy is greatly restrained by its low efficiency, the risk of inverted integration, and the allowable length of donor DNA template in certain delivery vectors, making it problematic for large dystrophin deletion mutations.

The homology-independent targeted integration (HITI), an NHEJ-based knockin approach, has no limitations regarding cell type, cellular proliferation status, and off-target integration [171,172]. This HITI technique uses an exogenous donor template containing the desired DNA sequence, which is flanked by the Cas9 cleavage sites. Once Cas9 protein cleaves both the genomic target sequence and the donor template, NHEJ repair machinery ensures the precise and efficient integration of a donor DNA sequence into the genomic locus. Notably, Cas9 protein repeatedly acts on the cleavage sites in the case of inverted integration until the occurrence of the desired insertion of the donor sequence. Recently, an HITI-mediated exon knockin strategy has been used to insert the missing human exon 52 in the hDMDΔ52/*mdx* mice (Figure 1E) [145]. The exogenous donor template lacks homology arms but contains either human exon 52 or the superexon encompassing the last 28 exons of the human *dystrophin* gene. Both intramuscular and systemic injections of the SaCas9 system and exogenous DNA template by AAV vectors have been found to effectively restore full-length dystrophin expression in the skeletal and cardiac muscles of hDMDΔ52/*mdx* mice. The restoration rate of cardiac dystrophin expression in systemically treated hDMDΔ52/*mdx* mice is 10% to 50% of the normal levels [145]. HITI-mediated superexon knockin approach has great potential for treating more than 20% of global DMD patients.

## 8. Base Editing

The base editing system, whose editing events do not rely on DSB generation and endogenous NHEJ machinery, offers a powerful strategy for safely correcting DMD mutations, especially point mutations [116]. Two major categories of DNA base editing tools exist: cytosine base editors (CBEs) catalyzing the C•G to T•A transitions, and adenine base editors (ABEs) converting the A•T to G•C base pairs [116,173]. Mechanistically, base editing-driven mutation corrections are accomplished either by direct base conversions at the mutational position or by exon skipping (Figure 1F). Through inducing G-to-A conversion at the splice site of a mutant or surrounding exon, the CBE fused between nSaCas9 and cytidine deaminase AID is employed to skip exon 50 in the cardiomyocytes from human DMD ΔEx51 iPSCs or trigger exon 4 skipping in the Dmd^E4^* mice harboring a 4-bp deletion within exon 4 [149,162]. The editing efficiency of this CBE in human DMD ΔEx51 iPSCs can be up to ~90% at the genomic level, which brings about nearly complete restorations of dystrophin and β-dystroglycan in the corrected cardiomyocytes [162]. The systemic administration of AAV9-encoded CBE and sgRNA in neonatal Dmd^E4*^ mice not only leads to the highly efficient restoration of dystrophin protein in cardiac and skeletal muscles but also prevents the onset of DMD symptoms throughout the mouse lifespan [149]. Because CBE-induced dystrophin restoration in the heart can be durable for at least 12 months, a single AAV9-CBE treatment is sufficient to extend the life span of corrected Dmd^E4*^ mice [149]. Since off-target editing events of CBEs have been described at both the genomic and transcriptomic levels [174,175,176], there is an urgent need to examine whether the in vivo application of CBEs may cause unpredictably detrimental outcomes (e.g., oncogenesis) in several DMD animal models.

It has been suggested that ABEs are safer than CBEs when rewriting the genome due to their high specificity and low off-target activity [176,177]. In support of their therapeutic potential, multiple ABE variants have been shown to be effective in DMD mice carrying single exonic deletion or nonsense mutations [146,147,148]. By directly introducing an A-to-G substitution at the point mutation site, the nSpCas9-ABE7.10 system has been found to cause genomic correction at ~3.3% efficiency and restore dystrophin expression in up to 17% of skeletal myofibers in DMD mice with a nonsense mutation in exon 20 [147]. Similarly, the systemic infusion of the nSpCas9-miniABE(GG) system in adult mdx^4cv^ mice leads to restored dystrophin expression, ameliorated muscle pathology, and improved muscle functions in both cardiac and skeletal muscles [148]. Genomic editing efficiency and therapeutic benefits can last for at least 9 months in the ABE-corrected mdx^4cv^ mice [148]. As an instance of ABE-triggered exon skipping, the nSpCas9-ABEmax system efficiently rescues the dystrophin reading frame in both human DMD ΔEx51 iPSCs and neonatal DMD ΔEx51 mice via inducing exon 50 skipping [146]. The local delivery of nSpCas9-ABEmax components into the skeletal muscles of DMD ΔEx51 mice can induce dystrophin restoration in nearly all skeletal myofibers and thereby prevent muscle pathology [146].

## 9. Prime Editing

Besides inducing the transitions of C•G to T•A and A•T to G•C as observed in BE systems, the versatile prime editing is capable of installing all other base substitutions, small insertions, and/or small deletions at the target locus. The prime editing system needs at least a prime editor fused between a reverse transcriptase and an nCas9 protein, and a prime editing guide RNA (pegRNA) [118,178]. PegRNA comprises a spacer complementary to the target site, an sgRNA scaffold, a primer binding site, and a reverse transcription template encoding the desired genomic sequence. Upon binding to the target site, the primer editor nicks the PAM-contained DNA strand, initiates reverse transcription, and synthesizes a new 3′ DNA flap containing the desired modification [118]. This newly synthesized 3′ DNA flap is eventually incorporated into the genome with the aid of endogenous DNA repair pathways. Since coordinating multiple pegRNA components is an essential prerequisite for precise genomic modification, prime editing is thought to trigger negligible byproduct events around the off-target sites.

The prime editing system has been demonstrated to rescue the dystrophin reading frame and protein expression in both human DMD ΔEx51 iPSCs-derived cardiomyocytes (Figure 1G) and human DMD myoblasts with an Ex6 mutant [146,163]. Prime editing-mediated insertion of two nucleotides within downstream exon 52 is found to reframe the ORF of dystrophin transcripts and generate a functional dystrophin protein reaching up to 39.7% of the normal level in the corrected cardiomyocytes [146]. Moreover, this reframing of exon 52 ameliorates abnormal calcium handling and improves the contractility in prime editing-corrected cardiomyocytes [146]. Given that the ΔEx51 mutation accounts for ~8% of all DMD patients [8], it will be valuable to assess the in vivo efficacy and durability of therapeutic prime editing in DMD animal models.

## 10. DMD Animal Models

More than 60 different animal models of DMD have been identified or generated in Caenorhabditis elegans, Drosophila, zebrafish, rodents, rabbits, dogs, pigs, and nonhuman primates [47,179,180,181,182,183,184,185,186,187,188,189,190]. These DMD animal models either naturally occur or are genetically engineered for the studies of disease mechanisms and clinical translation, and each animal model has its advantages and limitations. Although they are easily reproductive and relatively inexpensive, DMD rodent models generally exhibit mild clinical features of DMD patients owing to the complementary utrophin upregulation and their robust muscle regeneration capacity [191,192]. For example, the most frequently utilized *mdx* mice do not have moderate pathological signs until they are 15 months old and show just a 25% reduction in lifespan [179,180,181]. Unlike small rodent models, DMD pigs can develop severe disease phenotypes, but they die prematurely before breeding age [183,184], making them difficult to breed on a large scale. Conversely, DMD dogs, another typical large-animal model, display a 75% shortened lifespan similar to human DMD patients and can be bred relatively easily [185,193]. Meanwhile, canine DMD models closely resemble the disease progression and severity experienced by DMD patients, including limb muscle fibrosis and cardiomyopathy [64,65,194]. It seems that DMD dogs may be more suitable for preclinical translational studies than other large animal models. Nonetheless, their broad application in therapeutic translation is restrained by the heavy economic burden and the long time it takes to breed them in sufficient numbers [195]. Albeit both DMD rabbits and monkeys are already established via direct injection of SpCas9 and sgRNAs [186,187], they have not been applied in therapeutic testing due possibly to genetic mosaicism. It is important to note that a single animal model cannot fully mimic all pathological symptoms of human DMD patients. Given the unique characteristics of each DMD animal model, different animal models are proposed to recapitulate specific stages of human DMD progression [182,195]. Murine models are similar to the neonatal to the 3-year-old stage of DMD patients, the canine models represent the 5- to 10-year-old stage, and the porcine models resemble the later stage with cardiac defects. Therefore, it is necessary to insightfully consider and choose the optimal animal model for assessing therapeutic strategies.

## 11. Future Challenges and Prospects

### 11.1. Safety

The AAV-based delivery system is the most widely used vehicle for in vivo gene therapy in preclinical and clinical studies, mainly due to its high transduction efficiency, low immunogenicity, and durable therapeutic benefits [196,197]. Notably, the tissue tropism of multiple AAV serotypes (e.g., AAV6, AAV8, and AAV9) to skeletal and cardiac muscles makes these vectors particularly suitable for DMD gene therapy. Since mammalian muscle accounts for approximate 40% of total body mass [198], achieving durable and efficient genome editing in DMD animals requires high AAV vector doses, normally ranging from 5.5 × 10^14^ to 1.8 × 10^15^ vector genomes (vg)/kg [35,37,128,132]. This requirement of a high vector dosage poses formidable challenges for clinical-grade AAV manufacturing and the safety of AAV-based gene therapies. The single intravenous administration of high-dose AAV9 (at least 1.5 × 10^14^ vg/kg) could trigger notable adverse events in multiple organs, such as liver toxicity and kidney injury in dogs, piglets, and nonhuman primates [199,200]. In some clinical trials involving DMD patients, the systemic delivery of the AAV9 vector carrying the human micro-dystrophin gene at high doses (5 × 10^13^ to 3 × 10^14^ vg/kg) is reported to cause serious adverse events, including cardiopulmonary insufficiency and thrombocytopenia [201], which might be responsible for the recently described patient death. Therefore, it is crucial to determine the optimal vector dosage before the clinical application of systemic AAV therapeutics.

Given the packaging limitation of AAV vectors, the dual-AAV vector system is most often utilized for in vivo delivery of CRISPR gene-editing components. One major drawback of this dual-AAV system is the essential high dosage of AAV vectors for efficient gene editing. As an optimization strategy to reduce the viral dose, the self-complementary AAV (scAAV) vector with a double-strand viral genome has been developed to transport the sgRNA expression cassette into the skeletal and cardiac muscles of DMD ΔEx44 mice [136]. Unlike single-stranded AAV (ssAAV) vectors, the scAAV bypasses the rate-limiting second-strand synthesis and is resistant to degradation [202,203]. The dosage of scAAV to achieve an efficient genome modification is at least 20-fold lower than that of ssAAV [136]. Thus, the combination of sgRNA-expressed scAAV and Cas-packaged ssAAV may act as an attractive dual-vector system to ensure durable therapeutic efficacy in DMD individuals. However, there remains a need to further optimize the AAV delivery system and design all-in-one AAV vectors to accelerate the clinical translation of CRISPR gene therapy.

### 11.2. Immunogenicity

Another major concern of AAV-CRISPR gene therapy is the innate and adaptive immune responses evoked by AAV vectors and Cas proteins. Pre-existing anti-AAV antibodies are found in a large proportion of the human population [204,205], so some DMD patients with abundant AAV-neutralizing antibodies may be ineligible for AAV gene therapy. In this circumstance, either plasmapheresis or immunosuppressant needs to be administrated prior to AAV gene therapy for the reduction of anti-AAV antibody titer or the decline of immune system activity in the host patients [169,206]. Beyond the immunogenicity of AAV vectors, Cas-specific immune responses have been documented in murines, canines, and humans, owing to the bacterial and archaeal sources of Cas proteins [37,141,207,208,209]. For example, SaCas9- or SpCas9-specific antibodies and T cells have been ascertained in around 78% or 58–67% of healthy human populations [208]. On the contrary, the sgRNAs are merely reported to stimulate innate immune responses within human cells in vitro [210,211], but whether they are immunogenic in vivo remains to be determined. It is noteworthy that both cellular and humoral immune responses against AAV vectors and SaCas9 are not observed in neonatal mice after the systemic administration of AAV-SaCas9 components [37], suggesting that host immune responses to AAV vectors and Cas proteins might be avoided by treatment at the juvenile stage. This observation has been largely attributed to the lower essential AAV dosage, the more preserved muscles, and the absence of pre-existing immunity against AAV-CRISPR components at young ages [30,37]. Alternatively, the optimized forms of Cas9 proteins without immunogenic epitopes have been developed [212], and their in vivo application holds great potential to reduce the activity of the host immune system. Regardless, since immunosuppressant corticosteroids are normally used to dampen inflammation in DMD patients, one feasible solution to address the above immunogenicity is administrating corticosteroids with CRISPR gene therapy.

### 11.3. Off-Target Activity

The potential off-target activity of CRISPR systems poses an obstacle to their clinical application. In this regard, SpCas9 has been found to have a relatively high off-target activity owing to its tolerance of up to five mismatches between the guide sequence and target genome site [213,214,215]. Because most off-target cleavage events are present in highly proliferating cells in culture, the off-target genome editing of CRISPR systems is thought to be very low in animal models, especially in post-mitotic skeletal and cardiac cells [30,66]. Nonetheless, it cannot exclude the possible occurrence of deleterious off-target mutations in a specialized cell in vivo. Thus far, several different approaches have been developed to minimize such off-target mutagenesis. An attractive strategy is to use high-fidelity Cas enzymes (e.g., HypaCas9, evoCas9, SpCas9-HF1, and enAsCas12a-HF1) possessing high on-target specificity but low off-target activity [103,104,105,106,114,216]. It has been shown that Cas12 proteins generally exhibit much lower off-target editing activity than Cas9 variants [92,217], so their application is a relatively safe choice for CRISPR therapeutics. As for the second strategy, the sgRNAs can be elaborately optimized by either truncation or extension in a specific manner [218,219]. The truncated sgRNAs of less than 20 nucleotides in length can decrease genome-wide off-target events by up to five-fold [218]. In the third method, the utilization of muscle-specific promoters to specifically drive CRISPR component expressions in muscles can prevent the appearance of off-target editing events in undesirable tissues such as liver and kidney [128,138,220].

### 11.4. Durability

It should be noted that the skeletal muscles of both DMD patients and animals maintain high turnover rates throughout their lives [221,222]. As a result, dystrophin-positive myofibers following CRISPR therapeutics may be gradually diluted out of existence by dystrophic myofibers in the long term. Because muscle satellite cells undergoing de novo myogenesis are responsible for muscle regeneration [223,224], the delivery of CRISPR components into satellite cells could theoretically ensure durable therapeutic benefits in dystrophic muscles. Several studies have reported that systemic and intramuscular administrations of AAV9 vectors carrying SpCas9 or SaCas9 system in DMD mouse models induce Cas9 component expression and genomic editing in muscle satellite cells [127,225,226,227]. This achievement of satellite cell gene editing following systemic AAV9-CRISPR therapy can maintain restored dystrophin expression for 18 months in the skeletal muscles of *mdx* mice [225]. Therefore, the effective genome editing of muscle satellite cells is likely a valuable strategy for yielding lifelong therapeutic benefits, but its efficiency needs to be further improved. In the second strategy, micro-dystrophin is co-delivered with the SaCas9 system to achieve durable dystrophin restoration in skeletal muscles [129]. The systemic administration of the AAV6-encoded micro-dystrophin gene not only stabilizes skeletal myofibers but also halts CRISPR component loss, consequently allowing for persistent gene correction and ensuring lifelong skeletal dystrophin expression in DMD mdx^4Cv^ mice [129]. Considering the broad application of micro-dystrophin gene therapy in clinical trials, AAV-based co-delivery of micro-dystrophin and the CRISPR system shows great promise for the efficient treatment of diverse DMD patients.

## 12. Conclusions

The development and application of CRISPR-Cas technologies provide new opportunities for treating various genomic mutations at the source, enabling the durable restoration of protein expression and functions in the correct tissues. To date, the lifelong benefits of one-time CRISPR therapeutics have been manifested in some DMD mouse models at the preclinical level [35,37,43,44,148,149]. Although the in vivo therapeutic efficacy of CRISPR systems is promising without doubt, their safety profiles, especially concerning immunogenicity, AAV delivery and off-target issues, are imminently necessary to be addressed in several DMD animal models. As the challenges prior to clinical translation are overcome in the near future, the lessons from CRISPR therapeutics in DMD should apply to other devastating genetic diseases that lack effective therapies.

## Figures and Tables

**Table 1:** CRISPR-mediated therapeutic strategies in preclinical DMD animals.

Strategy	Mutation	Nuclease	Target Region	DMD Model	Delivery	Infusion	Reference
Double-cut exon exicision	Ex23 mut	SpCas9	i22, i23	*mdx* mice	AAV9	IM, IV, IP, RO	[34,35]
	Ex23 mut	SaCas9	i22, i23	*mdx* mice	AAV8	IM, IV, IP	[36,37]
	ΔEx52	SpCas9	i50, i51	DMDΔ52 mice	AAV9	IM, IV	[39]
	ΔEx52	SaCas9	Ex47, Ex58	hDMDΔ52/*mdx* mice	AAV9	IV	[40]
	Ex23 mut	SaCas9	i20, i23	*mdx* mice	AAVrh74	IV	[43]
	Dup Ex18-30	SaCas9	i21	Dup 18-30 mice	AAV9	IV	[125]
	Ex23 mut	SpCas9	i20, i23	*mdx* mice	AAV	IM	[126]
	Ex23 mut	SaCas9	i22, i23	*mdx* mice	AAV9	IM, IV, IP	[127]
	Ex53 mut	SaCas9, SpCas9	i51, i53	mdx^4Cv^ mice	AAV6	IM, RO	[128,129]
	ΔEx45	SpCas9	i44, i55	hDMDΔ45/mdxD2 mice	Plasmid	IM	[130]
	Ex23 mut	SaCas9	i20, i23	*mdx*/Utr^+/−^ mice	AAV	IV	[131]
Single-cut exon skipping and reframing	ΔEx44	SpCas9	Ex45	DMDΔ44 mice	AAV9	IM, IP	[132]
	ΔEx50	SaCas9-KKH	Ex51	DMDΔ50 mice	AAV9	IP	[133]
	ΔEx43	SpCas9	Ex44	DMDΔ43 mice	AAV9	IM	[134]
	ΔEx45	SpCas9	Ex44	DMDΔ45 mice	AAV9	IM	[134]
	ΔEx52	SpCas9	Ex53	DMDΔ52 mice	AAV9	IM	[134]
	ΔEx50	SpCas9-VRQR	Ex51	DMDΔ50;h51KI mice	AAV9	IP	[135]
	ΔEx44	SpCas9	Ex45	DMDΔ44 mice	AAV9	IP	[44,136]
	Ex23 mut	CjCas9	Ex23	DMD/Ex23 mut mice	AAV9	IM	[137]
	ΔEx50	SpCas9	Ex51	DMDΔ50 mice	AAV9	IM, IP	[138]
	ΔEx50	SpCas9	Ex51	ΔEx50-Dmd-Luc mice	AAV9	IM, IP	[139]
	ΔEx50	SpCas9	Ex51	DMDΔ50 dogs	AAV9	IM, IV	[140]
	Pseudo Ex13	SpCas9	i13	WCMD dogs	AAV8	IM, IV	[141]
	Pseudo Ex19	SpCas9	i19	LRMD dogs	AAV8	IM, IV	[141]
HDR-based correction	Ex23 mut	SpCas9	Ex23	*mdx* mice	Injection	Zygote	[124]
	Ex53 mut	SpCas9	Ex53	mdx^4Cv^ mice	AAV6	IM	[128]
	Ex23 mut	LbCas12a	Ex23	*mdx* mice	Injection	Zygote	[142]
	i6 mut	SpCas9	i6	GRMD dogs	Plasmid	IM	[143]
	Ex23 mut	SpCas9	Ex23	*mdx* mice	Nanoparticle	IM	[144]
Exon knockin	Ex51 mut	SaCas9	Ex52	hDMDΔ52/*mdx* mice	AAV9	IM, IV	[145]
Base editing	Ex51 mut	ABEmax-nSpCas9	Ex50	DMDΔ51 mice	AAV9	IM	[146]
	Ex20 mut	ABE-nSpCas9	Ex20	DMD/Ex20* mice	AAV9	IM	[147]
	Ex53 mut	ABE-nSpCas9-iNG	Ex53	mdx^4Cv^ mice	AAV9	IV	[148]
	Ex4 mut	CBE-nSaCas9	Ex4	Dmd^E4*^ mice	AAV9	IP	[149]

Ex, exon; i, intron; mut, mutation; AAV, adeno-associated viral vector; Δ, deletion; Dup, duplication; IM, intramuscular; IV, intravenous; IP, intraperitoneal; RO, Retro-orbital; KI, Knockin.

**Table 2:** CRISPR-mediated therapeutic strategies in human DMD cells.

Strategy	Mutation	Nuclease	Target Region	DMD Model	Delivery	Reference
Double-cut exon exicision	ΔEx46-51	SpCas9	i44, i55	human iPSCs	electroporation	[38]
	ΔEx52	SpCas9	i50, i51	human iPSCs	AAV6	[39]
	ΔEx49-50, ΔEx50-52, ΔEx51-53, ΔEx51-56	SaCas9	Ex47, Ex58	human myoblasts	lentivirus	[40]
	Dup Ex18-30	SpCas9	i27	human myoblasts	lentivirus	[122]
	Dup Ex55-59	SpCas9	i54	human iPSCs	nucleofection	[150]
	Dup Ex2	SpCas9	i2	human myoblasts	lentivirus	[151]
	Dup Ex3-16	SpCas9	i9	human myoblasts	lentivirus	[152]
	Dup Ex18-25	SpCas9	i25	human myoblasts	lentivirus	[153]
	ΔEx48-50	SpCas9	i50, i51	human myoblasts	electroporation	[154]
	ΔEx48-50	SpCas9	i44, i55	human myoblasts	electroporation	[154]
	ΔEx51	SpCas9, AsCas12a	i44, i55; i45, i54	human myoblasts	electroporation	[155]
	ΔEx45-52	SpCas9	i52, i53	human myoblasts	adenovirus	[156,157]
	ΔEx45-52, ΔEx48-50	SpCas9, eSpCas9(1.1)	i43, i54	human myoblasts	adenovirus	[156,157,158]
	ΔEx8-9	SpCas9	i2, i7; i5, i7; i6, i11	human iPSCs	nucleofection	[159]
	ΔEx3-7	SpCas9	i7, i9	human iPSCs	nucleofection	[159]
	Ex23 mut	SpCas9	i22, i23	mouse myoblasts	lipotransfection	[160]
Single-cut exon skipping and reframing	ΔEx44	SpCas9	Ex45	human iPSCs	nucleofection	[132]
	ΔEx48-50	SaCas9-KKH	Ex51	human iPSCs	nucleofection	[133]
	ΔEx43, ΔEx45	SpCas9	Ex44	human iPSCs	nucleofection	[134]
	ΔEx52	SpCas9	Ex51	human iPSCs	nucleofection	[134]
	ΔEx48-50	SpCas9-VRQR	Ex51	human iPSCs	nucleofection	[135]
	ΔEx48-50	LbCas12a, AsCas12a	Ex51	human iPSCs	nucleofection	[142]
	ΔEx48-50	SpCas9	Ex51	human iPSCs	nucleofection	[150]
	Pseudo Ex47	SpCas9	i47	human iPSCs	nucleofection	[150]
	ΔEx48-50	eSpCas9(1.1)	Ex51	human myoblasts	adenovirus	[158]
HDR-based knockin	i6 mut	SpCas9	i6	canine myoblasts	nucleofection	[143]
	ΔEx44	SpCas9	Ex44	human iPSCs	electroporation	[161]
Base editing	ΔEx51	ABEmax-nSpCas9	Ex50	human iPSCs	nucleofection	[146]
	ΔEx51	CBE-nSaCas9	Ex50	human iPSCs	lipotransfection	[162]
Prime editing	ΔEx51	PE2-dSpCas9	Ex52	human iPSCs	nucleofection	[146]
	Ex6 mut	PE2-nSpCas9	Ex6	human myoblasts	electroporation	[163]

Δ, deletion; Ex, exon; i, intron; iPSC, induced pluripotent stem cell; AAV, adeno-associated viral vector; Dup, duplication; mut, mutation.

## Data Availability

Not applicable.
